# Regional differences in clear cell metastatic renal cell carcinoma patients across the USA

**DOI:** 10.1007/s00345-023-04589-4

**Published:** 2023-09-27

**Authors:** Lukas Scheipner, Stefano Tappero, Mattia Luca Piccinelli, Francesco Barletta, Cristina Cano Garcia, Reha-Baris Incesu, Simone Morra, Andrea Baudo, Zhe Tian, Fred Saad, Shahrokh F. Shariat, Carlo Terrone, Ottavio De Cobelli, Alberto Briganti, Felix K. H. Chun, Derya Tilki, Nicola Longo, Luca Carmignani, Martin Pichler, Georg Hutterer, Sascha Ahyai, Pierre I. Karakiewicz

**Affiliations:** 1 Cancer Prognostics and Health Outcomes Unit, Division of Urology, University of Montréal Health Center, Montréal, QC Canada; 2 Department of Urology, Medical University of Graz, Auenbruggerpl. 1, 8036 Graz, Austria; 3 Department of Urology, IRCCS Policlinico San Martino, Genoa, Italy; 4 Department of Surgical and Diagnostic Integrated Sciences (DISC), University of Genova, Genoa, Italy; 5 Department of Urology, IEO European Institute of Oncology, IRCCS, Milan, Italy; 6 Università Degli Studi di Milano, Milan, Italy; 7 Unit of Urology/Division of Oncology, Gianfranco Soldera Prostate Cancer Lab, IRCCS San Raffaele Scientific Institute, Vita-Salute San Raffaele University, Milan, Italy; 8 Department of Urology, University Hospital Frankfurt, Goethe University Frankfurt am Main, Frankfurt am Main, Germany; 9 Martini-Klinik Prostate Cancer Center, University Hospital Hamburg-Eppendorf, Hamburg, Germany; 10 Department of Neurosciences, Science of Reproduction and Odontostomatology, University of Naples Federico II, 80131 Naples, Italy; 11 Department of Urology, IRCCS Policlinico San Donato, Milan, Italy; 12 Department of Urology, Comprehensive Cancer Center, Medical University of Vienna, Vienna, Austria; 13 Department of Urology, Weill Cornell Medical College, New York, NY USA; 14 Department of Urology, University of Texas Southwestern, Dallas, TX USA; 15 Hourani Center for Applied Scientific Research, Al-Ahliyya Amman University, Amman, Jordan; 16 Department of Oncology and Haemato-Oncology, Università degli studi di Milano, 20122 Milan, Italy; 17 Department of Urology, University Hospital Hamburg-Eppendorf, Hamburg, Germany; 18 Department of Urology, Koc University Hospital, Istanbul, Turkey; 19 Department of Urology, IRCCS Ospedale Galeazzi-Sant’Ambrogio, Milan, Italy; 20 Department of Oncology, Medical University of Graz, Graz, Austria; 21 Department of Hematology and Oncology, Medical Faculty, University of Augsburg, Augsburg, Germany

**Keywords:** SEER, Metastatic RCC, Regional, Outcome, Clear cell, Population

## Abstract

**Purpose:**

To test for regional differences in clear cell metastatic renal cell carcinoma (ccmRCC) patients across the USA.

**Methods:**

The Surveillance, Epidemiology, and End Results (SEER) database (2000–2018) was used to tabulate patient (age at diagnosis, sex, race/ethnicity), tumor (N stage, sites of metastasis) and treatment characteristics (proportions of nephrectomy and systemic therapy), according to 12 SEER registries. Multinomial regression models, as well as multivariable Cox regression models, tested the overall mortality (OM) adjusting for those patient, tumor and treatment characteristics.

**Results:**

In 9882 ccmRCC patients, registry-specific patient counts ranged from 4025 (41%) to 189 (2%). Differences across registries existed for sex (24–36% female), race/ethnicity (1–75% non-Caucasian), N stage (N1 25–35%, NX 3–13%), proportions of nephrectomy (44–63%) and systemic therapy (41–56%). Significant inter-registry differences remained after adjustment for proportions of nephrectomy (46–63%) and systemic therapy (35–56%). Unadjusted 5-year OM ranged from 73 to 85%. In multivariable analyses, three registries exhibited significantly higher OM (SEER registry 5: hazard ratio (HR) 1.20, *p* = 0.0001; SEER registry 7:HR 1.15, *p* = 0.008M SEER registry 10: HR 1.15, *p* = 0.04), relative to the largest reference registry (*n* = 4025).

**Conclusion:**

Important regional differences including patient, tumor and treatment characteristics exist, when ccmRCC patients included in the SEER database are studied. Even after adjustment for these characteristics, important OM differences persisted, which may require more detailed analyses to further investigate these unexpected differences.

## Introduction

Overall survival of clear cell metastatic renal cell carcinoma (ccmRCC) patients improved over the past decade [1, 2]. The introduction of new systemic therapies, specifically immunotherapies, has significantly contributed to this improvement [3]. However, most improvements were reported in the context of prospective randomized trials that may not apply to patients at large. It is possible that patient characteristics and patterns of care may differ between geographic regions of patient’s residence. Moreover, these differences could potentially lead to discrepancies in survival outcomes that should ideally not exist. Indeed, such differences across geographic regions have been reported for other urologic malignancies such as prostate or penile cancer [4–6]. It is currently unknown, whether such differences also exist for ccmRCC patients in the USA. We tested this hypothesis within the Surveillance, Epidemiology, and End Results (SEER) database (2000–2018). We hypothesized that such differences exist and that they may be associated with differences in overall mortality (OM) between specific geographic regions of residence (SEER registries).

## Methods

### Study population

The SEER database (2000–2018) was used to identify patients aged ≥ 18 years with histologically confirmed unilateral metastatic RCC (International Classification of Disease for Oncology [ICD-O] site codes C64.9), who harbored clear cell histology (ICD-O-3 code 8310). Cases identified only at autopsy were excluded. The SEER database is divided into 13 geographic registries. We excluded the smallest registry due to the limited sample size (*n* = 20). In accordance with the SEER data agreements and limitations, names of individual registries were omitted from the report [7]. These selection criteria resulted in an overall cohort of 9882 assessable patients within 12 SEER registries, namely from SEER registry 1 to SEER registry 12, in descending order of patient count. Death was defined according to the SEER mortality codes [8]. For the purpose of this study, OM (defined as death from any cause) was considered.

### Statistical analyses

Descriptive statistics included frequencies and proportions for categorical variables. Medians and interquartile ranges (IQR) were reported for continuously coded variables. Kruskall–Wallis rank sum and Pearson Chi-square tested for statistical significant differences in medians and proportions, respectively. Statistical analyses relied on three steps. First, baseline patient (age at initial diagnosis, sex, race/ethnicity: Caucasian vs. non-Caucasian), tumor (N stage according to the American Joint Committee on Cancer (AJCC) TNM system, 8th edition, sites of metastasis) and treatment (rate of nephrectomy (radical or partial) and systemic therapy) characteristics were tabulated and displayed graphically, according to the above defined SEER registries. Second, we relied on multinomial regression models to display adjusted proportions of nephrectomy and systemic therapy exposure. Here, multinomial models were fitted for each registry, and the adjusted treatment proportion was derived from the predicted probability of receiving the said outcome on the entire selected SEER population (including all registries) from the multinomial model of each registry. For proportions of nephrectomy, multinomial models relied on age, sex, year of diagnosis, race/ethnicity, as well as N stage as covariates. For systemic therapy exposure, multinomial models relied on age, sex, year of diagnosis, race/ethnicity, N stage, as well as nephrectomy proportions as covariates. Finally, 5-year OM was computed for each SEER registry. Moreover, unadjusted and adjusted OM hazard ratios (HR) were computed for each SEER registry, relying on Cox regression analyses. Adjustment variables consisted of year of diagnosis, baseline patient (age at diagnosis, sex, race/ethnicity), tumor (N stage, sites of metastasis) and treatment (nephrectomy and systemic therapy exposure) characteristics. All tests were two sided with a level of significance set at p < 0.05 and R software environment for statistical computing and graphics (version 4.1.2) was used for all analyses (7). Owing to the anonymously coded design of the SEER database, study-specific ethics approval was waived by the institutional review board.

## Results

### Descriptive characteristics

A total of 9822 ccmRCC patients were identified. Median age at initial diagnosis was 63 years (interquartile range (IQR) 56–71), 31% were female and 30% were non-Caucasians. N stage distribution was as follows: N0 6,085 (62%) vs. N1 3,192 (32%) vs. NX 605 (6%). Proportions of treatment were as follows: 5,666 patients (57%) received either radical (55%) or partial (2%) nephrectomy and 4,749 (48%) received systemic therapy (Table 1).

**Table 1:** Descriptive characteristics of 9882 clear cell metastatic renal cell carcinoma (ccmRCC) patients within the Surveillance, Epidemiology, and End Results (SEER) database (2000–2018)

Characteristic	*N*	Overall, *N* = 9882^1^
Age	9882	63 (56, 71)
Race	9882	
Caucasian		6948 (70%)
Non-Caucasian		1627 (30%)
N-Stage	9882	
N0		6690 (62%)
N1		3192 (32%)
NX		605 (6%)
Systemic therapy		4749 (48%)
Surgery	9863	5666 (57,3%)
Radical nephrectomy		5439 (55%)
Partial nephrectomy		227 (2.3%)
Female		3047 (31%)

### Differences in patient and tumor characteristics, across SEER registries

**Fig. 1:**
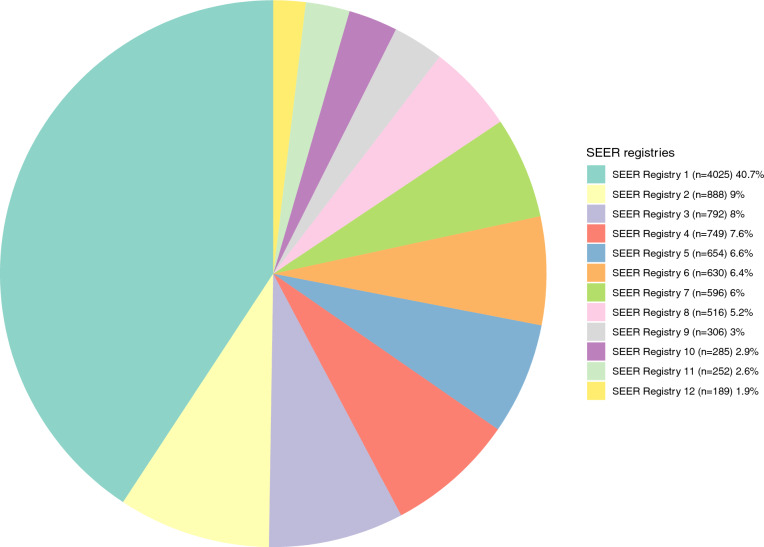
Pie chart depicting the distribution of 9882 clear cell metastatic renal cell carcinoma (ccmRCC) patients, according to the Surveillance, Epidemiology, and End Results (SEER) 2000–2018 geographic registries across the USA

**Fig. 2:**
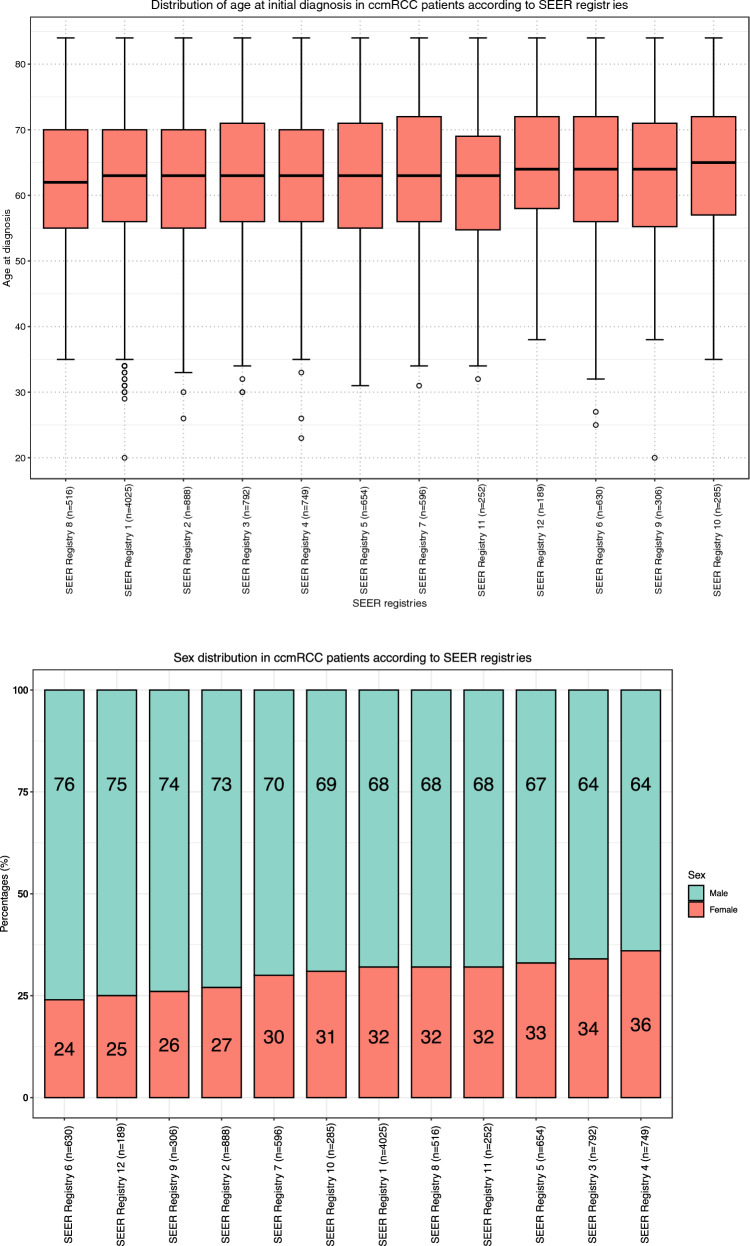

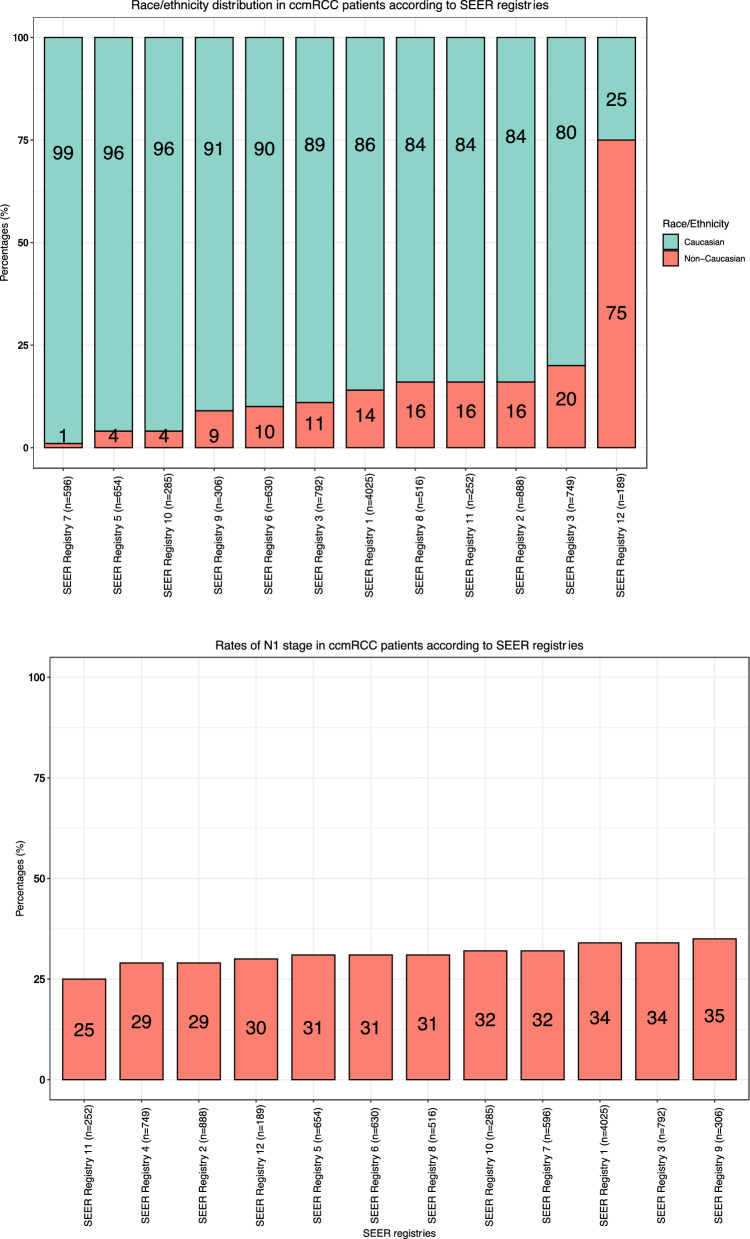

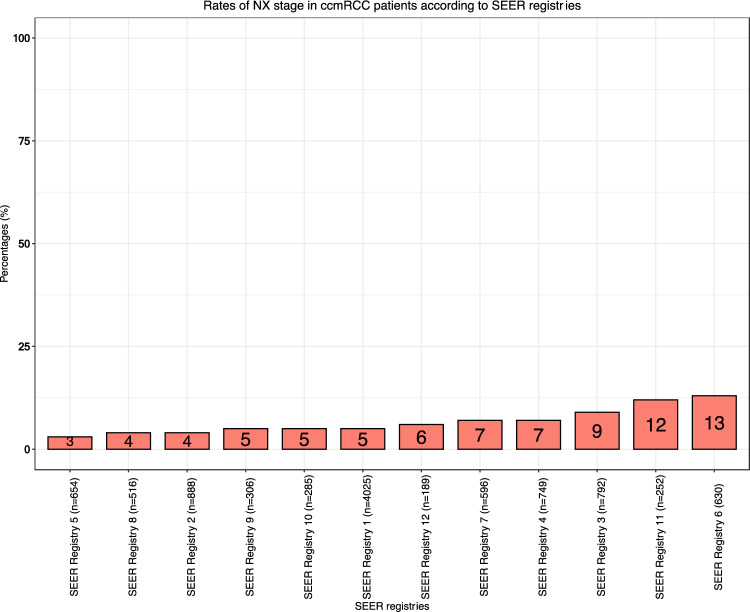
**a** Box and whisker plots depicting patient age at initial diagnosis distribution in 9882 clear cell metastatic renal cell carcinoma (ccmRCC) patients, according to the Surveillance, Epidemiology, and End Results (SEER) geographic registries (2000–2018). **b** Stacked bar plots depicting sex distribution in 9882 clear cell metastatic renal cell carcinoma (ccmRCC) patients, according to the Surveillance, Epidemiology, and End Results (SEER) geographic registries (2000–2018). **c** Stacked bar plots depicting race/ethnicity (Caucasian vs. Non-Caucasians) distribution in 9882 clear cell metastatic renal cell carcinoma (ccmRCC) patients, according to the Surveillance, Epidemiology, and End Results (SEER) geographic registries (2000–2018). **d** Stacked bar plots depicting rate of N1 stage in 9882 clear cell metastatic renal cell carcinoma (ccmRCC) patients, according to the Surveillance, Epidemiology, and End Results (SEER) geographic registries (2000–2018). **e** Stacked bar plots depicting rate of NX stage in 9882 clear cell metastatic renal cell carcinoma (ccmRCC) patients, according to the Surveillance, Epidemiology, and End Results (SEER) geographic registries (2000–2018)

Registry-specific patient counts ranged from 4025 (41%) in SEER registry 1 to 189 (2%) in SEER registry 12 (Fig. 1). The proportion of females ranged from 24 (SEER registry 6) to 36% (SEER registry 4; *p* < 0.001, Fig. 2b). The proportion of race/ethnicity other than Caucasians ranged from 1 (SEER registry 7) to 75% (SEER registry 12; *p* < 0.001; Fig. 2c). Regarding N stage, the proportions of N0 ranged from 56 (SEER registry 6) to 67% (SEER registry 2) vs. 25 (SEER registry 11) to 35% (SEER registry 9) for N1 (*p* < 0.001; Fig. 2d) vs. 3 (SEER registry 5) to 13 (SEER registry 6) for NX (*p* < 0.001; Fig. 2e). There were no statistically significant differences in patients’ age at initial diagnosis across the different SEER registries (*p* = 0.18; Fig. 2a). Regarding the location of metastasis, we recorded significant differences in the proportion of bone metastasis across the registries (21–31%; *p* < 0.001). No statistically significant differences were observed for the proportion of liver, lung, brain and other metastasis across the SEER registries (Table 2).

**Table 2:** Patterns of metastasis of 9882 clear cell metastatic renal cell carcinoma (ccmRCC) patients according to Surveillance, Epidemiology, and End Results (SEER) 2000–2018 geographic registries across the USA

Characteristic	Overall, *N* = 9882^a^	SEER registry 1 (*n* = 4025)	SEER registry 2 (*n* = 888)	SEER registry 3 (*n* = 792)	SEER registry 4 (*n* = 749)	SEER registry 5 (*n* = 654)	SEER registry 6 (*n* = 630)	SEER registry 7 (*n* = 596)	SEER registry 8 (*n* = 516)	SEER registry 9 (*n* = 306)	SEER registry 10 (*n* = 285)	SEER registry 11 (*n* = 252)	SEER registry 12 (*n* = 189)	*P* value^b^
Bone	2443 (25%)	926 (23%)	255 (29%)	180 (23%)	201 (27%)	204 (31%)	141 (22%)	163 (27%)	127 (25%)	65 (21%)	77 (27%)	52 (21%)	52 (28%)	< 0.001
Liver	1005 (10%)	392 (9.7%)	115 (13%)	75 (9.5%)	89 (12%)	62 (9.5%)	51 (8.1%)	61 (10%)	58 (11%)	37 (12%)	21 (7.4%)	24 (9.5%)	20 (11%)	0.062
Lung	3990 (40%)	1609 (40%)	346 (39%)	306 (39%)	306 (41%)	275 (42%)	257 (41%)	241 (40%)	211 (41%)	116 (38%)	137 (48%)	100 (40%)	86 (46%)	0.3
Brain	770 (7.8%)	326 (8.1%)	70 (7.9%)	50 (6.3%)	50 (6.7%)	50 (7.6%)	36 (5.7%)	59 (9.9%)	38 (7.4%)	25 (8.2%)	20 (7.0%)	26 (10%)	20 (11%)	0.13
Other	976 (9.9%)	391 (9.7%)	83 (9.3%)	75 (9.5%)	67 (8.9%)	85 (13%)	64 (10%)	66 (11%)	42 (8.1%)	28 (9.2%)	25 (8.8%)	33 (13%)	17 (9.0%)	0.2

^a^Median (IQR); n (%)

^b^Kruskal–Wallis rank sum test; Pearson's Chi-square test

### Unadjusted and adjusted differences in treatment proportions across SEER registries

**Fig. 3:**
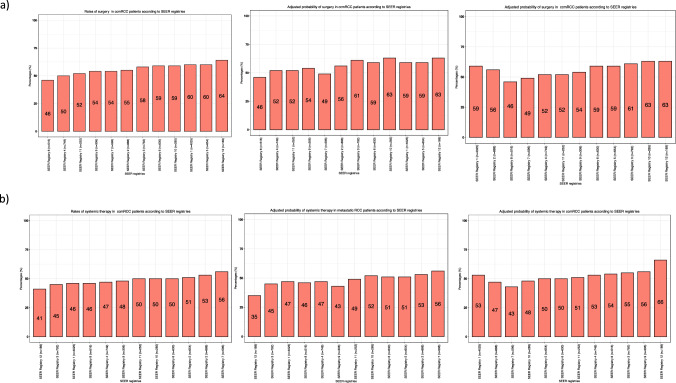
Bar plots depicting rates of **a** surgery and **b** systemic therapy before and after multinomial adjustment in in 9882 clear cell metastatic renal cell carcinoma (ccmRCC) patients, according to the Surveillance, Epidemiology, and End Results (SEER) geographic registries (2000–2018). The first plot shows SEER registries in ascending order according to rates of treatment before adjustment, the second plot shows SEER registries in the same order after adjustment and the third plot shows the largest two SEER registries (1,2) and then the smallest in ascendant order after adjustment

The rate of nephrectomy ranged from 46 (SEER registry 8) to 64% (SEER registry 12; *p* < 0.001, Δ = 18%). After adjustment, differences in nephrectomy proportions persisted (46–63%, Δ = 17%, *p* < 0.001). When focusing on the two registries with the highest patient count (SEER registry 1–2), proportions ranged from 56 to 59% (Δ = 3%). In the ten remaining registries with lower patient count (SEER registry 3 to 12), proportions ranged from 46 to 63% (Δ = 17%; Fig. 3a). Rate of systemic therapy ranged from 41 (SEER registry 12) to 56% (SEER registry 7; *p* < 0.001, Δ = 15). After adjustment, differences in systemic therapy proportions persisted (35–56%, Δ = 21, *p* < 0.001). When focusing on the two registries with the highest patient count (SEER registry 1–2), proportions ranged from 47 to 53% (Δ = 6%). In the ten remaining registries with smaller patient count (SEER registry 3 to 12), proportions ranged from 35 to 56 (Δ = 21%; Fig. 3b).

### Overall mortality and unadjusted and adjusted differences in overall mortality across SEER registries

Five-year OM was 80% for all 9882 ccmRCC patients. Five-year registry-specific OM ranged from 73 to 85% (Table 3). When focusing on the two registries with the highest patient count (SEER registry 1–2), OM ranged from 79 to 80% (Δ = 1%). In the ten remaining registries with lower patient count, 5-year OM ranged from 73 to 85% (Δ = 12%). Unadjusted HR predicting OM ranged from 0.93 to 1.17. Adjusted HR predicting OM ranged from 0.88 to 1.20. The HR predicting OM recorded in three registries was statistically significantly higher than the recorded HR of SEER registry of reference (SEER registry 1, HR 1.0). Specifically, the HR recorded for SEER registry 5 was 1.20 (*p* = 0.0001), the HR recorded for SEER registry 7 was 1.14 (*p* = 0.008) and the HR for SEER registry 10 was 1.15 (*p* = 0.04; Table 3).

**Table 3:** Overall mortality (OM) in 9882 clear cell metastatic renal cell carcinoma (ccmRCC) patients according to Surveillance, Epidemiology, and End Results (SEER) 2000–2018 geographic registries across the USA

SEER Registries	1 (*n* = 4025)	2 (*n* = 888)	3 (*n* = 792)	4 (*n* = 749)	5 (*n* = 654)	6 (*n* = 630)	7 (*n* = 596)	8 (*n* = 516)	9 (*n* = 306)	10 (*n* = 285)	11 (*n* = 252)	12 (*n* = 189)
Five-year OM (%)	79.1	80.1	78.1	83.5	84.8	78.8	83.1	84.5	77.4	86.3	72.5	79.1
Unadjusted OM HR	Ref	1.05	0.97	**1.15**	**1.13**	0.97	**1.14**	**1.17**	0.97	**1.17**	0.93	0.97
95% CI***		(0.96–1.14)	(0.88–1.05)	**(1.05–1.26)**	**(1.02–1.24)**	(0.88–1.07)	**(1.03–1.25)**	**(1.05–1.29)**	(0.84–1.11)	**(1.01–1.33)**	(0.79–1.08)	(0.81–1.14)
*p* value		0.25	0.48	**0.001**	**0.01**	0.60	**0.008**	**0.003**	0.67	**0.02**	0.34	0.68
Adjusted OM HR*	Ref	1.08	1.00	1.09	**1.20**	0.96	**1.14**	1.03	0.91	**1.15**	0.88	0.97
95% CI***		(0.99–1.17)	(0.91–1.09)	(0.98–1.19)	**(1.09–1.32)**	(0.87–1.06)	**(1.03–1.26)**	(0.93–1.15)	(0.79–1.04)	**(1.01–1.32)**	(0.75–1.02)	(0.81–1.16)
*p* value		0.07	0.91	0.06	**0.0001**	0.48	**0.008**	0.48	0.18	**0.04**	0.10	0.76

Bold values indicate* p*-value < 0.05

*Adjusted for sex, age, systemic therapy, race/ethnicity, N stage, sites of metastasis, year of diagnosis and surgery

**p value < 0.05

***Confidence Intervals

## Discussion

It is currently unknown whether regional differences regarding patient, tumor and treatment characteristics exist in ccmRCC patients and potentially even contribute to differences in overall mortality (OM). We hypothesized that higher than expected OM may be identified in select SEER registries, even after adjustment for patient, tumor and treatment characteristics. We tested this hypothesis within a large population of ccmRCC patients from within the SEER database (2000–2018). Our analyses resulted in several noteworthy observations.

First, we identified 9882 ccmRCC patients of 12 geographic registries within the SEER database over a period of 18 years (2000–2018). This number is comparable to a different study addressing ccmRCC within the SEER database over a similar time period [9]. Analyses on regional differences regarding patient, tumor and treatment characteristics as well as cancer control outcomes, as were done in this study, require use of large-scale population-based databases. Single-institution or even multi-institutional databases may suffer from deficient numbers of observations or patient populations, which limits this type of research. In consequence, large-scale epidemiologic databases such as SEER or the National Cancer Database (NCDB) are essential for the purpose of assessing regional differences in patient, tumor or treatment characteristic as well as OM outcomes in ccmRCC patients.

Second, we recorded important differences in patient, tumor and treatment characteristics between the SEER registries. Regarding patient characteristics, the proportions of female patients ranged from 24 to 36% (*p* < 0.001) and the proportion of race/ethnicity other than Caucasians ranged from 1 to 75% across the SEER registries (*p* < 0.001). Proportions of N1 stage ranged from 25 to 35% and proportions of unknown N stage (NX) ranged from 3 to 13% (*p* = 0.008; Fig. 2d). In a recent National Cancer Database (NCDB) analysis, female sex was an independent predictor for worse OS in ccmRCC [10]. Similarly, a SEER-based analysis reported that non-Caucasians experience higher CSM in ccmRCC compared to Caucasians [11]. Last but not least, N1 status has been shown to be an independent predictor for worse CSM [12]. Regarding treatment characteristics, proportions of nephrectomy ranged from 46 to 64% (*p* < 0.001). These differences persisted after adjustment for age, sex, year of diagnosis, race/ethnicity and N status (46–63%). Moreover, we observed marginal variability in the two registries with the highest patient count (Δ = 3%). Conversely, the recorded variability between the ten registries with smaller patient count was more pronounced (Δ = 17%). Cytoreductive nephrectomy plays an integral role in the management of ccmRCC; however, its indication depends on multiple clinical variables and ultimately on an individualized clinician’s assessment. In consequence, its use may vary and its variability may not be directly related to tumor characteristics. Additionally, we observed important differences in systemic therapy exposure, ranging from 41 to 56% across the SEER registries (p < 0.001). These differences persisted after adjustment (35–56%). Systemic therapy represents the key element in multimodal treatment of ccmRCC. The presence of such differences in systemic therapy exposure may potentially affect survival rates. Furthermore, all the above-mentioned registry-specific differences may result in OM outcome discrepancies. Therefore, it is crucial to include these patient, tumor and treatment characteristics in multivariable analyses addressing OM, as was done in the current analyses.

Third, we also identified important variability in registry-specific five-year OM ranging from 72.5% (SEER registry 11) to 84.5% (SEER registry 8). Additionally, unadjusted OM HR was significantly higher in five registries with lower patient count compared to the registry of reference (SEER registry 1) with the highest patient count: SEER registry 4 HR 1.15, SEER registry 5 HR 1.13, SEER registry 7 HR 1.14, SEER registry 8 HR 1.17 and SEER registry 10 HR 1.17. However, these rates may be biased, due to differences in patient, tumor and treatment characteristics. In consequence, we reassessed these rates after detailed multivariable adjustment. Despite this extensive adjustment, HR differences persisted (ranging from 0.88 to 1.20). Specifically, the OM HR remained significantly higher in three registries with lower patient count (SEER registry 5: HR 1.20, p = 0.0001; SEER registry 7: HR 1.14; *p* = 0.008; SEER registry 10: HR 1.15, *p* = 0.04). Taken together, these results indicate that only three out of twelve regions exhibit suboptimal survival data. Ideally, no statistically significant differences should be recorded after adjustment for patient case mix. Interestingly, these registries represent registries with lower patient count. In consequence it is possible that a systematic disadvantage may exist in smaller SEER registries. The structure of the SEER database does not allow investigating in more detail the specific association between low patient counts and worse survival. However, it is well established that according to the practice-makes-perfect hypothesis, small caseload and lack of regionalization tend to be associated with worse outcomes including worse survival [13]. In consequence, regionalizing the care for ccmRCC patients may represent a valid option for avoiding low patient counts at regional or institutional level. Regionalization of care, as well as standardization of care, in addition to multidisciplinary decision making at larger centers, all have the ability to improve survival, as well as all other outcomes.

Despite the novelty of the current study, our work has limitations and should be interpreted in the context of its retrospective and population-based design. First, the current SEER version provides sampling of patient from only 12 specific registries. This sample may not perfectly reflect the entire US population. Additionally, since the SEER database is designed with the intent of providing a representation of the US population, our findings cannot be applicable to patients from other countries and should be ideally validated after adjustment for ccmRCC characteristics using large-scale database in multi-collaborative studies even in other countries or macro-areas. Third, the SEER database does not allow stratifying or adjusting the analyses, according to the International Metastatic Database Consortium (IMDC) criteria. However, this limitation applies to all previous SEER and NCDB analyses. Fourth, limited details regarding treatment type is available. Specifically, the SEER database does provide information on systemic therapy. Therefore, a distinction between chemotherapies and immunotherapies is not possible, nor does it provide information on cycle number and duration of treatment administration. Fifth, multivariable adjustment relies on patient, tumor and treatment information available in the SEER database. It is possible that other unavailable patient, tumor and treatment characteristics also affected the observed rates, without being amendable for inclusion in either stratification or multivariable adjustment. Unfortunately, the SEER database does not provide data regarding baseline comorbidity status. Ideally, it could have been used for the purpose of further adjustment.

## Conclusion

Important regional differences including patient, tumor and treatment characteristics exist, when ccmRCC patients included in the SEER database are studied. Even after adjustment for these characteristics, important OM differences persisted, which may require more detailed analyses to further investigate these unexpected differences.

## Data Availability

All data generated for this analysis were from the Surveillance, Epidemiology, and End Results Research Plus (SEER) database. The code for the analyses will be made available upon request.
